# A phase I dose-escalating study of DaunoXome, liposomal daunorubicin, in metastatic breast cancer

**DOI:** 10.1038/sj.bjc.6600344

**Published:** 2002-07-15

**Authors:** K J O'Byrne, A L Thomas, R A Sharma, M DeCatris, F Shields, S Beare, W P Steward

**Affiliations:** University Department of Oncology, Osborne Building, Leicester Royal Infirmary, Leicester LE1 5WW, UK

**Keywords:** metastatic breast cancer, liposomal daunorubicin, DaunoXome

## Abstract

The aims of this phase I study were to establish the maximum tolerated dose, safety profile and activity of liposomal daunorubicin, DaunoXome (NeXstar Pharmaceuticals), in the treatment of metastatic breast cancer. DaunoXome was administered intravenously over 2 h in 21 day cycles and doses were increased from 80 to 100, 120 and 150 mg m^2^. Sixteen patients were enrolled. A total of 70 cycles of DaunoXome were administered. The maximum tolerated dose was 120 mg m^2^, the dose-limiting toxicity being prolonged grade 4 neutropenia or neutropenic pyrexia necessitating dose reductions at 120 and 150 mg m^2^. Asymptomatic cardiotoxicity was observed in three patients: grade 1 in one treated with a cumulative dose of 800 mg m^2^ and grade 2 in two, one who received a cumulative dose of 960 mg m^2^ and the other a cumulative dose of 600 mg m^2^ with a previous neoadjuvant doxorubicin chemotherapy of 300 mg m^2^. Tumour response was evaluable in 15 patients, of whom two had objective responses, six had stable disease and seven had progressive disease. In conclusion, DaunoXome is associated with mild, manageable toxicities and has anti-tumour activity in metastatic breast cancer. The findings support further phase II evaluation of DaunoXome alone and in combination with other standard non-anthracycline cytotoxic or novel targeted agents. Although the dose-limiting toxicity for DaunoXome was febrile neutropenia at 120 mg m^2^, we would recommend this dose for further evaluation, as the febrile neutropenia occurred after four or more cycles in three of the four episodes seen, was short lived and uncomplicated.

*British Journal of Cancer* (2002) **87**, 15–20. doi:10.1038/sj.bjc.6600344 www.bjcancer.com

© 2002 Cancer Research UK

## 

The anthracyclines, doxorubicin and epirubicin, used as single agents or in combination regimens, are established as first line treatment in the management of locally advanced and metastatic breast cancer (A'hern *et al*, 1993). The use of these drugs is, however, limited by their acute toxicities and propensity to cause cumulative cardiac damage (Buzdar *et al*, 1985). Liposomal encapsulation of anti-cancer drugs is a novel therapeutic strategy in the management of patients with malignant disease (Langer, 1998). Potential advantages of liposome-mediated drug delivery systems include increased plasma drug concentrations, improved drug delivery and tumour targeting, reduced toxicity and evidence of increased efficacy (Gabizon, 1992). These contentions are supported by recent large phase II and III studies of Caelyx™ (pegylated stealth liposomal doxorubicin, Doxil, Sequus Pharmaceuticals, Menlo Park, CA, USA) in patients with solid tumours. In a study of 71 patients with metastatic breast cancer the agent was well tolerated with a reduction in alopecia, nausea and vomiting, and neutropenic pyrexia compared to that expected from conventional doxorubicin therapy. No significant cardiotoxicity was seen with the Caelyx™ treatment. This improvement in tolerability was not at the expense of anti-tumour activity, the objective response rate being 31% (Ranson *et al*, 1997). Furthermore, Caelyx™ has enhanced anti-tumour activity compared to conventional doxorubicin in relapsed ovarian cancer and has been demonstrated to be at least equivalent in efficacy to topotecan (Gordon *et al*, 2001).

Daunorubicin is an anthracycline which has been used in the treatment of leukaemias since the 1960s. Daunorubicin has been evaluated in the treatment of a wide variety of solid tumours with objective responses being seen in relatively large phase II studies of soft tissue sarcoma and colorectal cancer. Tumour responses have also been noted in non-small cell and small cell lung, renal and prostate cancer, choriocarcinoma and neuroblastoma, and with intravesical treatment of bladder cancer. In the 1980s, development of daunorubicin in the treatment of solid tumours including breast cancer was discontinued due to the tremendous number of anthracycline analogues and anthracene derivatives (e.g. mitoxantrone) being evaluated (Harvey *et al*, 1984; Von Hoff, 1984).

DaunoXome is a liposome-encapsulated form of daunorubicin in which the anthracycline has been entrapped in small unilamellar vesicles (diameter: 40–80 nm, mean: 45 nm) composed of a 2 : 1 molar ratio of highly purified distearoyl phosphatidylcholine (DSPC) and cholesterol. The pharmacokinetic profile of DaunoXome is different from conventional daunorubicin with a 36-fold increase in the area under the plasma concentration curve and a first phase elimination half-life of 5.3 to 8.3 h (Rahman *et al*, 1984a; Forssen and Ross, 1994; Gill *et al*, 1995; Bellott *et al*, 2001). *In vivo* experiments indicate increased uptake of daunoXome in tumour tissue at 24 h compared to conventional daunorubicin (Forssen *et al*, 1992, 1996). DaunoXome (NeXstar Pharmaceuticals) is licensed for the treatment of AIDS-related Kaposi's sarcoma (Gill *et al*, 1995, 1996). Given the early encouraging anti-tumour activity of daunorubicin, DaunoXome was evaluated in a phase I study in solid tumours. The recommended phase II dose for patients who had received prior chemotherapy was found to be 100 mg m^2^ and in chemotherapy-naïve individuals, 120 mg m^2^ (Guaglianone *et al*, 1994). In a study in relapsed or refractory lymphomas, one complete and two partial responses were seen in nine patients treated at 120 mg m^2^ (Richardson *et al*, 1997). Apart from significant neutropenia (grade 3 or higher) seen at doses of 80 mg m^2^ or above, the agent appears well tolerated with a reduction in alopecia, nausea and vomiting and perhaps also cardiotoxicity compared to standard anthracyclines (Guaglianone *et al*, 1994; Richardson *et al*, 1997).

There is little data on the use of daunorubicin in the treatment of breast cancer. Van Hoff (1984) identified only four patients with breast cancer treated in early phase studies in whom no anti-tumour activity was observed. Subsequently clear evidence emerged that not all anthracyclines had equal efficacy in metastatic breast cancer. For example idarubicin, esorubicin and carcinomycin had inferior response rates to doxorubicin and/or epirubicin (Rozencweig *et al*, 1984; Bonfante *et al*, 1986; Lopez *et al*, 1989). However, *in vitro* studies indicate that daunorubicin has equivalent cytotoxicity to doxorubicin in breast adenocarcinoma lines (Wiles *et al*, 1997). Furthermore, a preliminary report presented by Hupperets *et al* (1996) indicated that DaunoXome 100 mg m^2^ every 21 days for up to 24 weeks had encouraging single agent anti-tumour activity in metastatic breast cancer with objective tumour responses in three and stable disease in seven out of 11 treated patients (Anonymous, 1996). This evidence was deemed appropriate to justify a phase I study of DaunoXome in an anthracycline-naïve population presenting with metastatic breast cancer.

## PATIENTS AND METHODS

### Study design

A phase I dose-escalating study was developed to establish the MTD of, and to obtain preliminary efficacy and tolerability data for, DaunoXome in the treatment of anthracycline-naïve patients with advanced breast cancer. A starting dose of 80 mg m^2^ was chosen because of the significant neutropenia reported at this level in previous work (Guaglianone *et al*, 1994).

Cohorts of at least three patients were to be treated at each dose level. The planned dose escalation was from 80 to 100, 120, 150 and 180 mg m^2^. No intra-patient dose escalation was permitted. For safety reasons, the next dose level was not opened until the final patient in a given cohort had been observed for 21 days. The dose-limiting toxicities (DLT) were defined as (i) any grade 3 or 4 non-haematologic drug-induced toxicity excluding alopecia, nausea or vomiting, (ii) any haematologic toxicity necessitating a dose reduction and (iii) any drug-induced adverse event that warranted removal of the patient from the study. If a DLT was encountered at any dose level, a further three patients were enrolled at that dose. If a DLT occurred in two patients then that dose level was declared the maximum tolerated dose (MTD) and no further dose escalations occurred.

### Eligibility criteria

Patients with evaluable, histologically or cytologically confirmed, metastatic breast cancer were eligible for the study. Prior non-anthracycline based chemotherapy, hormonal manipulation, immunotherapy and radiotherapy were permitted. Adjuvant or neoadjuvant anthracycline-based chemotherapy was allowed provided that the time to relapse after completion of chemotherapy was >6 months, the cumulative doxorubicin dose was ⩽300 mg m^2^ and epirubicin ⩽400 mg m^2^. Normal cardiac function, with left ventricular ejection fraction (LVEF) >50% as assessed by echocardiography was essential. Other inclusion criteria included Eastern Cooperative Oncology Group performance status of ⩽2, life expectancy of at least 12 weeks, adequate bone marrow function (total white cell count >3×10^9^ per l, absolute neutrophil count >1.5×10^9^ per l, haemoglobin count >9 g dl^−1^, and platelet count >100×10^9^ per l), adequate hepatic function (total bilirubin concentration <1.5×ULN, transaminases <3×ULN, unless due to liver metastases), adequate renal function (serum creatinine in the normal range) and no other primary cancer within 5 years (except basal cell skin carcinoma or carcinoma-*in-situ* of the cervix). Exclusion criteria included prior bone marrow transplantation, CNS involvement, concomitant radiotherapy, a history of cardiac disease or uncontrolled hypertension. Patients gave written informed consent to participation in the study, which was approved by the local Ethics Committee.

### Study treatment

DaunoXome was administered on day 1 of a 21 day cycle as a 2 h infusion in the out-patient department. Prophylactic anti-emetic treatment with 5HT-antagonists was not given. In the event of drug hypersensitivity, the infusion was continued with oral antihistamines for a mild reaction, and the rate of infusion could be slowed based on symptoms (grade 1). For a moderate or severe reaction (grades 2 or 3), the infusion was stopped, intravenous antihistamine and dexamethasone given, and the infusion restarted at a slower rate with premedication with antihistamine and steroids on further cycles. A maximum of eight cycles of DaunoXome could be administered. Prophylactic use of colony-stimulating growth factors was not permitted.

### On-study investigations

Pre-treatment evaluation included full blood count, renal, liver and bone biochemistry, ECG, echocardiography and establishment of evaluable disease sites by physical examination and/or radiology. On-treatment evaluation included a weekly full blood count, renal, liver and bone biochemistry profile prior to each treatment, echocardiography after four cycles (after two cycles if the patient had received prior adjuvant anthracycline-based therapy) and subsequently after every two cycles until the end of treatment.

Dose modification was carried out in the event of haematological or other toxicities as defined by the NCIC CTG Expanded Common Toxicity Criteria. If the full blood count was not adequate on day 21 (ANC >1.5×10^9^ per l or platelets >100×10^9^ per l), the treatment was deferred by 7 days. For grade 4 neutropenia persisting for >7 days, a dose delay >2 weeks, grade 4 thrombocytopenia or an episode of thrombocytopenic bleeding, the dose was reduced by 20 mg m^2^ or one dose level. In the event of grade 3 or 4 non-haematological toxicity (except for alopecia, and nausea and vomiting subsequently controlled by anti-emetics) the dose was again reduced by 20 mg m^2^ or one dose level.

In patients with easily accessible lesions, biopsies were performed 24 h after administration of the first cycle of DaunoXome to look for evidence of uptake of the agent into tumour tissue using confocal laser microscopy.

### Response evaluation

Tumour response was formally assessed after every two cycles of chemotherapy in accordance with WHO guidelines. A complete response was defined as the complete resolution of all disease. A partial response was defined as a reduction of 50% or more in the sum of the bi-dimensional mass measurements. Both responses required a duration of more than 4 weeks with a confirmatory physical examination and/or radiological assessment. Stable disease was defined as less than 50% reduction in the disease and progressive disease was defined as ⩾25% increase in the sum of the bi-dimensional tumour measurements. Duration of response was defined from the day of commencement of chemotherapy to the time of clinical evidence of disease progression.

## RESULTS

### Patient characteristics

Sixteen patients were enrolled onto the study. The median age was 51 years (range 34 to 68 years). Twelve of the 16 patients had visceral disease. The lungs, liver and bone were the most common sites of metastasis. Only one patient had not received prior treatment for breast cancer. Twelve patients had received adjuvant radiotherapy and eight had received adjuvant chemotherapy of which one had been anthracycline containing. Twelve patients had received prior hormonal therapy and in seven patients, second line hormonal therapy had been commenced at the time of diagnosis of metastatic disease. Additional patient characteristics are listed in Table 1

**Table 1:**
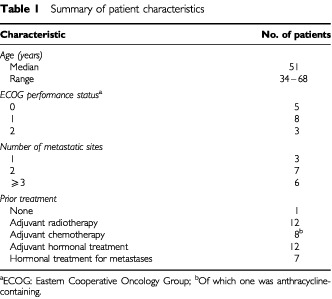
Summary of patient characteristics

. All patients were evaluable for toxicity. Altogether 70 cycles of treatment were given. The median number of cycles of DaunoXome administered was six per patient (range 1–8). Considering the 120 and 150 mg m^2^ dose levels of DaunoXome only, the mean number of cycles administered remained high at 5.75 per patient.

### Haematological toxicity

Grade 4 neutropenia was encountered in 11, and grade 3 in three patients (Table 2

**Table 2:**
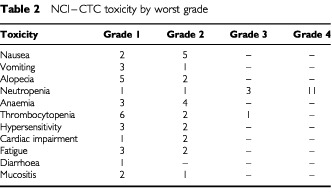
NCI–CTC toxicity by worst grade

). In the majority of cases the neutropenia was experienced during the first cycle of therapy without obvious cumulative myelosuppression. Neutropenia was complicated by a total of four febrile episodes affecting three patients. The febrile episodes occurred during cycle 1 of level 3 treatment in one patient only. This patient had a further febrile episode on the sixth cycle at level 2. The other febrile episodes occurred on the fourth cycle in one patient on level 4 and after six cycles in another on level 3. All episodes responded promptly to intravenous antibiotics. Eight out of the 70 cycles of chemotherapy were delayed due to neutropenia. As a result of these complications, dose reductions were required on five occasions (Table 3

**Table 3:**
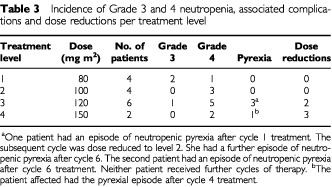
Incidence of Grade 3 and 4 neutropenia, associated complications and dose reductions per treatment level

). No grade 3 or 4 anaemia was seen. One patient experienced grade 3 thrombocytopenia but no episodes of thrombocytopenic bleeding were seen. The haematological toxicities defined the MTD at 120 mg m^2^ (Table 3).

### Cardiotoxicity

Pretreatment echocardiographic assessment revealed gross cardiac function to be normal in all patients with LVEF >50%. A significant asymptomatic change in cardiac function was seen in three patients: One at dose level 4 had grade 1 cardiac toxicity with a DaunoXome cumulative dose (CD) administered of 800 mg m^2^. An initial echocardiographic study revealed normal cardiac function but failed to give an accurate LVEF. She had subsequent studies with MUGA scans, that revealed deterioration in LVEF from 65 to 50% after six cycles of therapy. MUGA scans 1 and 3 months post-treatment revealed no further deterioration in cardiac function with LVEF of 51% on both occasions. One patient at dose level 2 with grade 2 cardiotoxicity received a DaunoXome CD of 600 mg m^2^ and had previously received a doxorubicin CD of 300 mg m^2^ in the adjuvant setting. Baseline LVEF=75% was unchanged after two and four cycles of treatment (74%) but fell to 46% after six cycles. Repeat studies 2, 5 and 8 months after the last administration of DaunoXome revealed no further deterioration in cardiac function with LVEF readings of 48, 53 and 53% respectively. A further patient at dose level 3 received a cumulative dose of 960 mg m^2^ and had a deterioration in LVEF from 84 to 57% over eight cycles of treatment with no further deterioration when evaluated 3 months later. She died 18 months after completion of DaunoXome therapy without any history of a cardiac event.

### Other non-haematological toxicity

No grade 3 or 4 non-haematological toxicity was seen (Table 2). The most common non-haematological toxicity was alopecia with seven of the 16 patients having some hair loss; this, however, was usually minimal with only two patients having grade 2 alopecia. Prophylactic anti-emetics with 5HT antagonists were not prescribed and there was very little nausea and vomiting seen; seven patients had grade 1 and 2 nausea and four had grade 1 and 2 vomiting. Five patients experienced a mild hypersensitivity reaction during treatment, the most common symptom being the onset of low back pain which was relieved by slowing the rate of DaunoXome infusion.

### Anti-tumour activity

Of the 15 patients evaluable for response, one achieved a complete response, one had a partial response, six had stable disease and seven had progressive disease. The overall median time to progression was 5 months (range 15–485+ days) which increased to 8.5 months (range 29–485+ days) at dose levels 3 and 4 (DaunoXome 120 and 150 mg m^2^). The overall median survival was 13.75 months (range 18–705 days). In the patients treated at dose levels 3 and 4, the median survival was 19 months.

## DISCUSSION

In keeping with the results of previous phase I and II studies in solid tumours and lymphomas, and with the *in vitro* data, this study indicates that DaunoXome is well tolerated and, at doses ⩾100 mg m^2^, has anti-tumour activity in metastatic breast cancer. The agent has a different toxicity profile to conventional anthracyclines and Caelyx™ (Buzdar *et al*, 1985; A'hern *et al*, 1993; Ranson *et al*, 1997). Unlike conventional anthracyclines, significant alopecia was not observed and the patients did not require prophylactic anti-emetics with 5HT antagonists, no grade 3 or 4 nausea or vomiting being seen. Plantar-palmar erythrodysesthesia, an epithelial toxicity observed in patients receiving protracted infusions or repeated dose-intensive administration of doxorubicin (Samuels *et al*, 1987; Bronchud *et al*, 1989), and the dose-limiting side-effect of caelyxs™ (Ranson *et al*, 1997), was not observed with DaunoXome in this study. This difference is most likely explained by the relatively protracted elimination half-life of Caelyx™ (half-life >48 h) compared with that of DaunoXome (half-life 5.3–8.3 h).

Grade 4 neutropenia was documented in 11 patients although febrile neutropenia only occurred in three, all of whom entered the study at either the 120 or 150 mg m^2^ dose levels. The febrile episodes were short-lived and uncomplicated. Out of the 70 cycles of chemotherapy delivered, only eight were delayed due to neutropenia. Five dose reductions were necessitated because of either protracted (>7 days) or pyrexia associated neutropenia, of which three were in the two patients initially treated at the 150 mg m^2^ dose level and the other two in one patient treated initially at 120 mg m^2^. These results indicate that the MTD in patients with metastatic breast cancer, treated without colony stimulating growth factor support, was DaunoXome 120 mg m^2^. As the pyrexial episodes were relatively uncomplicated, we would recommend this dose for evaluation in phase II studies. Other significant haematological complications were rarely seen with only one patient having grade 3 thrombocytopenia.

The level of myelosuppression was higher than that seen for Caelyx™ in breast cancer in which, in the largest reported study, no episodes of febrile neutropenia were seen (Ranson *et al*, 1997). This may in part be explained by the number of cycles the patients received. In the Caelyx™ study, a median of 3.6 cycles were administered to each patient which was significantly lower than the six per patient treated with DaunoXome in this study. Febrile neutropenia occurring in cycle 1 was seen in one patient only. The other episodes occurred after four or more cycles of therapy were given. Furthermore the mucocutaneous toxicity in the Caelyx™ study necessitated dose reductions and deferments, which may make the rate of grade 3/4 myelosuppression seen in the Caelyx™ study, and hence associated febrile complications, relatively lower.

Current recommendations indicate that CD of conventional daunorubicin should not exceed 650 mg m^2^ (Lenaz and Page, 1976). However the risk of cardiotoxicity falls if relatively low bolus doses of any given anthracycline are used allowing higher CDs to be administered (Sallan and Clavell, 1984). In animal models, the cardiotoxic effects of anthracyclines are ameliorated when administered in liposomal formulations (Rahman *et al*, 1984b). In this study, grade I cardiotoxicity was seen in one patient at level 4 and grade II in one patient at level 3, who received a DaunoXome CD of 800 and 960 mg m^2^ respectively. These patients did not have any obvious risk factors for developing cardiotoxicity and they had not received prior anthracycline chemotherapy. A further patient with grade 2 cardiotoxicity received daunoXome 100 mg m^2^ to a CD of 600 mg m^2^ but had received doxorubicin CD of 300 mg m^2^ in her combination adjuvant chemotherapy regimen. None of the patients required treatment. The reduction in LVEF stopped after completion of chemotherapy with no further deterioration in function either symptomatically or on imaging for up to 18 months following the last administration of DaunoXome. Therefore evidence of subclinical cardiotoxicity developed at CD levels one would anticipate might lead to symptomatic cardiac problems using conventional daunorubicin. A number of recent studies evaluating DaunoXome in the treatment of lymphomas and solid tumours, and using similar doses to those reported here, have reported an absence of cardiac side-effects (Fossa *et al*, 1998; Richardson *et al*, 1997).

That DaunoXome may be less cardiotoxic than conventional daunorubicin is supported by the recent observations for Myocet which encapsulates doxorubicin in a conventional liposome (The Liposome Company, Elan Corporation). Myocet has been shown to improve the therapeutic index of doxorubicin by significantly reducing cardiotoxicity, allowing higher CD of doxorubicin to be administered (Batist *et al*, 2001). Further work would be required to definitively establish the CD for DaunoXome infusion bolus doses ⩾100 mg m^2^ that should not be exceeded in order to prevent symptomatic cardiac toxicity. This is potentially of great importance given the toxicity profile of the recently developed therapeutic anti-HER2/neu monoclonal antibody, Herceptin, when used in combination with the conventional anthracycline doxorubicin. Despite enhancing the anti-tumour efficacy of cytotoxic agents and prolonging survival, significantly increased cardiac toxicity was seen in the anthracycline containing arm of the study (Slamon *et al*, 2001). Combination of herceptin with liposomal anthracyclines may represent a strategy to overcome this problem.

The study confirms that DaunoXome does have anti-tumour activity in metastatic breast cancer. A further recent study in metastatic breast cancer has reported objective tumour responses in three of nine patients treated with the agent (Darskaia *et al*, 1999). Combining the results of this study with the two published reports DaunoXome has shown objective responses in eight out of 35 (23%) evaluable patients (Anonymous, 1996; Darskaia *et al*, 1999). The response rate for this relatively non-toxic anthracycline based therapy must be taken in context with those reported for single-agent doxorubicin which vary from 30–34% in recent randomised phase III studies (Henderson *et al*, 1989; Sledge *et al*, 1997; Chan *et al*, 1999; Norris *et al*, 2000). The overall time to tumour progression of 5 months (8.5 months in those patients treated at 120 and 150 mg m^2^) and median survival of 13.75 months are in keeping with the results reported for large randomised studies of doxorubicin when used alone or as part of a combination regimen in the treatment of symptomatic relapsed breast cancer (Norris *et al*, 2000).

A series of phase I studies have evaluated the combination of Caelyx™ with the novel agents gemcitabine, vinorelbine and paclitaxel with encouraging response rates and acceptable toxicity profiles (Burstein *et al*, 1999; Schwonzen *et al*, 2000; Rivera *et al*, 2001). In a recent phase I study, DaunoXome was substituted for doxorubicin in the conventional CHOP regimen to treat patients with low or intermediate non-Hodgkin's lymphomas. Complete and partial responses were seen in 44% of treated patients and the MTD was 70–80 mg m^2^ depending on the population treated (Flinn *et al*, 2000). These results indicate the potential for inclusion of DaunoXome in combination regimens for metastatic breast cancer.

The anti-tumour efficacy and tolerability of DaunoXome demonstrated here make it eminently suitable for the treatment of metastatic breast cancer, where agents with minimal toxicity are often sought, particularly in elderly, frail patients. A randomised phase II study comparing DaunoXome with conventional doxorubicin has been proposed to compare anti-tumour activity, time to tumour progression, survival and quality of life. The agent also requires evaluation in phase II combination studies with conventional cytotoxic agents and novel targeted therapies such as Herceptin.
